# Diet in Disease

**Published:** 1893-03-04

**Authors:** Ernest Hart

**Affiliations:** Bachelier-ès-Sciences-es-Lettres (rèstreint), formerly Student of the Faculty of Medicine of Paris, and of the London School of Medicine for Women


### Diet in Disease.

By Mrs. Ernest Hart, Bachelier-ès-Sciences-es-Lettres (rèstreint), formerly Student of the Faculty of Medicine of Paris, and of the London School of Medicine for Women.

### XIV.—DIABETES (*continued*).

In order to facilitate the duties of the cook and housekeeper in providing for a diabetic patient an agreeable dietary, from which starch and sugar have been excluded, I have arranged a series of *ménus* for the day's meals, and will give, in many instances, the recipes for the dishes. It will be noticed in studying these *ménus* that four principles have been followed—firstly, to exclude starch and sugar; secondly, to supply their place by the hydro-carbon-fat, so that there may not be a lack of energy-producing and fat-forming food; thirdly, to make the meals digestible, a weakened digestion being a frequent accompaniment of diabetes; and fourthly, to make the food as appetising as possible. Thus, with these objects in view, it will be seen that cream is used in place of milk, cream being practically free from lactose, or sugar of milk; unripe fruits sweetened with saccharin take the place of ripe fruits; Bonthron's almond biscuits grated are used in thickening soups and sauces instead of arrowroot, and almond flour is employed instead of wheaten flour. Fish and vegetables are cooked with a liberal allowance of butter, and every opportunity is taken of adding the necessary amount of fat by means of such dainties as *foie gras*, cream-cheese, olives, &c. In order to make the food digestible, directions are given to Warrenise instead of to boil, and to braise instead of to bake. It will thus, I trust, be seen from these *ménus* that it is quite unnecessary to add to the miseries already endured by a diabetic that of a repulsive and unpalatable diet. A common-sense combination of science and the culinary art will produce for him as dainty dishes as any epicure may desire.

### A WEEK'S *MENUS* FOR A DIABETIC.

(*Time—Summer.*)

**First Day.**

*Breakfast.*

Buttered eggs.
Sole, fried in butter, with lemon juice added when served.
Cocoa made from nibs, with cream,
and "torrefied bread." (1)

*Lunch.*

Hot sardines on toasted gluten bread. (3)
Warrenised breast of lamb, with spring cabbage. (2)
Camembert cheese with Callard's biscuits.

*Dinner.*

Spinach soup. (4)
Cutlets of salmon fried in slippers.
Poulet a l'estragon. (5)
Green-gooseberry fool (6), sweetened with saccharin.

*Recipes.*

(1) Torrefied Bread is made by toasting thin slices of ordinary bread before the fire until they are deeply and thoroughly browned, almost blackened, so that the starch and gluten are in great part destroyed by the heat.—*Yeo.*

(2) Warrenised Breast of Lamb, With Spring Cabbage.—A Warren cooking pot is a very necessary *article de cuisine*. It is a pot consisting of three stages connected by a steam chimney. A small amount of water is put in the bottom of the pot; in the second stage the meat is placed with its flavourings, and in the top the vegetables. The food is, it will be seen, thus cooked by steam; all the juices of the meat are therefore retained, and not lost in the water as in boiling. Meat is rendered much more succulent, tender, and digestible by Warrenising than by boiling.

(3) Gluten Bread cut into slices, soaked in butter and toasted or fried, is very palatable, and will be found a useful article in the preparation of food for diabetics.

(4) Spinach Soup is made from a weak meat or bone stock, to which a fine purée of spinach is added. Some cream is added when the soup is poured into the tureen. Purée soups made of the vegetables permitted are very useful additions to the dietary. Among them may be mentioned turnip, tomato, sorrel, lettuce, and asparagus soups, to all of which cream may be added with advantage if it is well tolerated by the patient.

(5) Poulet a l'Estragon.—It will be found useful to study the various ways of preparing fowls from French and English cookery books, the forbidden ingredients being replaced by those permitted. The amended receipt can then be written out by the housekeeper and given to the cook for her guidance. Poulet a l'Estragon is a very palatable dish. Before cooking, the liver is removed and a bunch of fresh tarragon is placed inside the fowl. The fowl is then roasted or braised. When finished it is cut into joints which are placed upon croutons of gluten bread, the whole being sprinkled with chopped leaves of fresh tarragon. Fresh roasted tomatoes are placed round the dish. The liver and giblets are stewed with tarragon leaves. When sufficiently cooked the liver is rubbed through a fine hair sieve to thicken and flavour the gravy, which is served in a sauce boat.

(6) Green-gooseberry Fool.—The deprivation of ripe fruits is often severely felt by the diabetic patient. It is, however, perfectly safe for him to take unripe fruits before the sugar is developed in them, and these can be made into palatable and digestible dishes by stewing them with saccharin, passing them through a sieve, as in "fools," mixing cream into them, or by stewing them with saccharin.

**Second Day.**

*Breakfast.*

Fresh haddock fried in butter.
Cold tongue.
Coffee and cream.

*Lunch.*

Vegetable marrow farcie. (7)
Devilled ham and French beans. (8)
Cheddar cheese with diabetic biscuits and butter.

*Dinner.*

Oysters.
Clear soup.
Roast lamb.
Green asparagus with clear melted butter.
Almond pudding. (9)

*Recipes.*

(7) Vegetable marrow or cucumber make an excellent dish boiled and stuffed with veal force-meat, in which, instead of bread-crumbs or flour, Bonthron's almond biscuits must be used, but the force-meat must be bound together with a beaten egg.

(8) "Devils" are easily made, and render a dish of cold meat palatable and savoury. A paste is made of almond flour, curry powder, mustard, salt, and oil, with sauces to vary the flavour. This is spread on the cold meat to be devilled, before grilling. Served hot.

(9) Almond Pudding and Cakes.—The correct making of almond puddings and almond cakes by the cook of a diabetic is an art to be practised and mastered. When sweetened with saccharin they make tasty, sweet dishes, which prevent the patients from missing and longing for the forbidden puddings of former days. The following recipes will be found most valuable:—

Almond Pudding.—Take two eggs, a quarter of a pound of almond flour, and a quarter of a pound of butter, three tabloids of saccharin dissolved in a tablespoonful of brandy. Warm the butter, beat in the almond flour and the yolks of the eggs, adding the dissolved saccharin. Whisk the whites into a stiff froth, beat all together. Put into dariole moulds and bake in a quick oven, and serve with a little hot sauce made with dry sherry and saccharin.

Almond Biscuits.—To every ounce of almond flour add two whites of eggs and a little salt to taste. Beat the whites of eggs to a stiff froth, add the almond flour, and beat well together. Put in buttered patty-pans, and bake in a moderately quick oven from fifteen to twenty minutes. The whole has to be done quickly, and baked directly the ingredients are mixed. This biscuit will be found very useful as a substitute for bread.

**Third Day.**

*Breakfast.*

Fresh herrings with mustard sauce.
Savoury omelette.
Tea with cream.

*Lunch.*

Cold mutton with French bean salad mixed with oil and a dash of vinegar.
Stewed lettuce. (10)
Rochefort cheese with diabetic rusks.

*Dinner.*

Tomato soup.
Sweetbreads aux fonds d'artichauts. (11)
Fillet of beef garnished with cauliflowers.
Custard pudding sweetened with saccharin.

*Recipes.*

(10) Stewed Lettuce.—A well-grown lettuce is selected. It is first boiled in plenty of water, care being taken not to let it drop to pieces. When nearly done take out, drain, and place in a stew-pan with a little rich brown gravy, and allow it to simmer for twenty minutes.

(11) The Sweetbreads are first stewed in milk, then removed and rolled in slices of fat bacon and placed in the oven for a quarter of an hour. The bacon is then removed, and the sweetbreads are cut in slices and grated Parmesan cheese is shaken over them. They are again placed in the oven and braised in a rich brown glaze. Served on a crouton of gluten bread, on the centre is placed the *fonds d'artichauts* boiled and cut in quarters.

Fourth Day.

*Breakfast.*

Curried eggs (without rice). (12)
Ham.
Cocoa made from nibs, with cream.

*Lunch.*

Braised knuckle of veal with mixed vegetables (13)
Foie gras with diabetic biscuits.

*Dinner.*

Cock-a-leekie soup.
Turbot with tartar sauce.
Duck with olives.
Cucumber au sauce Fairlawn. (14)

*Recipes.*

(12) In making Curries, cocoanut or green apples can be used as the basis of the curry.

(13) The braising of meats makes them much more digestible and also more savoury than roasting. Put in the braising pot a little fat or butter and finely-chopped onion, and brown the knuckle of veal in it. Then add more fat—bacon fat being preferable—a few vegetables, spices, a bunch of herbs, salt, and pepper. Close the pot securely so as not to let the steam escape, and place hot coals on the li[illegible] from time to time to obtain equal heat top and bottom. Time taken half as long again as for roasting.

(14) Cooked Cucumber is a very useful article. It is boiled in the same way as vegetable marrow. "Sauce Fairlawn" is made from butter, and milk, and yolks of eggs, adding three table-spoonfuls of grated Parmesan before serving. This sauce is poured over the cucumber in the dish when served.

Fifth Day.

*Breakfast.*

Eggs, with black butter.
Grilled kidneys and bacon.
Cream and aerated water.

*Lunch.*

Fish pudding. (15)
Cold meat and tomato salad.
Neufchatel cream cheese and almond biscuits.

*Dinner.*

Bisque soup. (16)
Boiled fowl, with bechamel sauce (17) and baked mushrooms, and vegetable marrow.
Green currant fool.
Hot caviare and gluten croutons.

*Recipes.*

(15) Fish Pudding.—Make a thick white sauce of butter milk and yolks of eggs, to which either anchovy, Worcester, and Harvey sauces, ketchup, a little chopped anchovy, shredded onion, and a small amount of pickled mango are added according to taste. Pour the sauce over the fish after this has been broken up, and bake in a dish in the oven.

(16) Bisque Soup.—This is made in the usual way, except that it is thickened with almond biscuits grated instead of rice.

(17) In the Bechamel Sauce the beaten yolks of two or more eggs are added to thicken.

Sixth Day.

*Breakfast.*

Poached eggs and spinach.
Smoked salmon.
Van Houten's cocoa, made with cream.

*Lunch.*

Crab omelette. (18)

Cold or hot mutton.

Asparagus.

*Dinner.*

Sorrel soup.
Cream of veal.
Turkey poult, with French beans.
Cauliflower au gratin.

*Recipes.*

(18) Crab Omelette.—Break the eggs required into a basin, season with salt, pepper, chopped parsley, and a small piece of chopped shalot; beat well together with a whisk, shred the crab, and mix it with the eggs. Fry in butter in the usual way. Another way is to make the omelette and put the shredded crab inside instead of folding it over.

All kinds of omelettes excepting sweet omelettes—viz., omelettes with fine herbs, with kidneys, with oysters, with ham, &c., are suitable for diabetic patients.

Seventh Day.

*Breakfast.*

Kippered herrings.
Grilled bones, with buttered broccoli.
Egg flip. (19)

*Lunch.*

Mayonnaise of lobster.
Stewed pigeons with mushrooms.
Cauliflowers.
Gruyère cheese.

*Dinner.*

Julienne soup.
Sole broiled with white wine.
Grilled mutton cutlets with savoury sauce. (20)
French beans.
Lemon sponge.

*Recipes.*

(19) Egg Flip.—This will be found most useful, especially in those cases of diabetes where there is much dyspepsia, from which the patient suffers particularly in the morning, and is consequently unable to eat a good breakfast. Heat half a pint of milk not quite to boiling point; pour it on to the well-beaten yolk of an egg, stirring all the time. Add two table-spoonfuls of unsweetened whisky or brandy.

(20) Grilled Cutlets are much improved by a good sauce. The following recipe is excellent: Melt a piece of butter on a plate, and add a piece of glaze about the same size as the butter, also a little Harvey, Worcester, anchovy, or ketchup sauce, varying to taste. Well mix with a knife, and spread over the cutlets before broiling. When done, serve with the gravy from the chops.

